# *Toxoplasma gondii* Lysine Acetyltransferase GCN5-A Functions in the Cellular Response to Alkaline Stress and Expression of Cyst Genes

**DOI:** 10.1371/journal.ppat.1001232

**Published:** 2010-12-16

**Authors:** Arunasalam Naguleswaran, Eliana V. Elias, Jeanette McClintick, Howard J. Edenberg, William J. Sullivan

**Affiliations:** 1 Department of Pharmacology & Toxicology, Indiana University School of Medicine, Indianapolis, Indiana, United States of America; 2 Department of Biochemistry and Molecular Biology, Indiana University School of Medicine, Indianapolis, Indiana, United States of America; Weill Medical College of Cornell University, United States of America

## Abstract

Parasitic protozoa such as the apicomplexan *Toxoplasma gondii* progress through their life cycle in response to stimuli in the environment or host organism. Very little is known about how proliferating tachyzoites reprogram their expressed genome in response to stresses that prompt development into latent bradyzoite cysts. We have previously linked histone acetylation with the expression of stage-specific genes, but the factors involved remain to be determined. We sought to determine if GCN5, which operates as a transcriptional co-activator by virtue of its histone acetyltransferase (HAT) activity, contributed to stress-induced changes in gene expression in *Toxoplasma*. In contrast to other lower eukaryotes, *Toxoplasma* has duplicated its GCN5 lysine acetyltransferase (KAT). Disruption of the gene encoding for TgGCN5-A in type I RH strain did not produce a severe phenotype under normal culture conditions, but here we show that the TgGCN5-A null mutant is deficient in responding to alkaline pH, a common stress used to induce bradyzoite differentiation *in vitro*. We performed a genome-wide analysis of the *Toxoplasma* transcriptional response to alkaline pH stress, finding that parasites deleted for TgGCN5-A fail to up-regulate 74% of the stress response genes that are induced 2-fold or more in wild-type. Using chromatin immunoprecipitation, we verify an enrichment of TgGCN5-A at the upstream regions of genes activated by alkaline pH exposure. The TgGCN5-A knockout is also incapable of up-regulating key marker genes expressed during development of the latent cyst form, and is impaired in its ability to recover from alkaline stress. Complementation of the TgGCN5-A knockout restores the expression of these stress-induced genes and reverses the stress recovery defect. These results establish TgGCN5-A as a major contributor to the alkaline stress response in RH strain *Toxoplasma*.

## Author Summary

Protozoan parasites cause significant disease in humans and livestock, and many of our current therapies have serious side effects or are being rendered useless due to the development of drug resistance. These parasites typically have complex life cycles involving multiple hosts and some, like *Toxoplasma gondii*, have the ability to remain in the host for life as a latent tissue cyst. *Toxoplasma* is one of the most successful parasites on Earth because the ability to develop into a tissue cyst greatly facilitates transmission through carnivores. Cyst formation also is responsible for recrudescent infection in immunocompromised patients. The conversion of *Toxoplasma* from its replicating cell to the cyst is triggered by stress, but we have little understanding of how the parasite stress response functions. In this study, we identify the genes involved in *Toxoplasma*'s response to alkaline stress, which is a known trigger of cyst development. We also establish that a lysine acetyltransferase enzyme called TgGCN5-A is required for type I RH strain *Toxoplasma* to respond normally to alkaline stress. Parasites lacking TgGCN5-A are no longer capable of activating genes induced during cyst formation triggered by alkaline pH.

## Introduction

Stress responses are critical to cell survival, allowing cells to adapt to changing environmental conditions. In certain pathogenic eukaryotes, such as the protozoan *Toxoplasma gondii* (phylum Apicomplexa), the stress response takes on added significance as it triggers a developmental change into a latent cyst form. Parasitic protozoa often rely on stimuli in the environment or host organism in order to progress through the parasite life cycle. The study of stress-induced developmental changes in *Toxoplasma* is significant as this process underlies pathogenesis. This obligate intracellular protist develops from a rapidly growing form (tachyzoite) into a latent cyst form (bradyzoite) in response to stress [1]. In human hosts, the cyst forms can re-emerge as destructive tachyzoites if immunity wanes, causing recurring bouts of toxoplasmosis that may endanger immunocompromised individuals [2]. A major gap in our knowledge that impedes the development of novel therapeutics against *Toxoplasma* infection is our poor understanding of how tachyzoites reprogram their expressed genome in response to stresses that prompt cyst development. The identification of proteins that contribute to stress response and bradyzoite formation would be a significant step towards the design of new therapies to treat toxoplasmosis.

How the parasite coordinates the changes in gene expression that accompany stress-induced bradyzoite development is not clear, but epigenetic mechanisms, including histone modifications, have been implicated as contributing to this process [3], [4]. Formerly referred to as histone acetyltransferases (HATs), lysine acetyltransferases (KATs) of the general control nonderepressible-5 (GCN5/KAT2) family are well-conserved among eukaryotes [5]. While invertebrates generally possess a single GCN5, vertebrate species harbor two: GCN5 and the highly similar homologue called PCAF (p300/CBP-Associating Factor) [6]. The GCN5 KAT family has been implicated in cell-cycle progression [7], chromatin remodeling at specific promoters [8], transcription elongation [9], cellular differentiation [10], and the cellular stress response [11]. Microarray analyses of knockouts made in yeast suggest that GCN5 is a gene-specific coactivator, regulating 1.1% of genes in *Schizosaccharomyces pombe* and up to 4% in *Saccharomyces cerevisiae* [12], [13]. The GCN5 deletion mutant in *S. cerevisiae* is viable, but grows poorly on minimal media [14]. Similarly, GCN5 is not essential for growth under normal conditions in *S. pombe*, but is required for mounting an appropriate response to KCl and CaCl_2_-mediated stresses [15], [16]. In *Arabidopsis* plants, GCN5 controls ∼5% of genes and is important for normal growth and development, as well as the response to cold stress [17]. GCN5 was shown to be instrumental in the control of specific morphogenetic cascades during developmental transitions in *Drosophila* [18]. GCN5-null mouse embryos fail to form dorsal mesoderm lineages due to a high incidence of apoptosis and die 10.5 days post conception, suggesting a critical role for GCN5 in mammalian development [10], [19], [20]. In contrast, PCAF appears to be dispensable in mice as its loss confers no distinct phenotype [10]. Collectively, these studies support the idea that GCN5 KATs modulate gene expression during stress, or exposure to other environmental stimuli, to elicit the appropriate response or developmental change.

*Toxoplasma* is unique among fellow apicomplexan parasites and other invertebrates in possessing two GCN5 HATs, designated TgGCN5-A and –B [21], [22]. We sought to delineate the function of TgGCN5-A by creating a genetic knockout using homologous recombination in haploid RH strain tachyzoites. As in other lower eukaryotes, *Toxoplasma* lacking TgGCN5-A (ΔGCN5-A) showed no discernible phenotype under normal culture conditions [21], but its response to stress was not addressed. Here, we analyzed wild-type and ΔGCN5-A parasites under normal and alkaline pH growth conditions using *Toxoplasma* genome microarrays. The results illuminate the parasite's response to alkaline pH and demonstrate that TgGCN5-A is required for most of these changes in gene expression. We also show that ΔGCN5-A parasites exhibit greater sensitivity to alkaline pH stress - a novel role for GCN5 KATs. Moreover, *Toxoplasma* lacking TgGCN5-A cannot activate bradyzoite-specific genes that are normally up-regulated during alkaline pH-induced cyst development. These studies establish a novel function for GCN5 KATs in the eukaryotic response to alkaline stress and support the idea that TgGCN5-A is a key contributor to gene expression pertinent to the development of the latent cyst form of *Toxoplasma*.

## Results/Discussion

### Differential gene expression in *Toxoplasma* during alkaline pH stress

**Figure 1:**
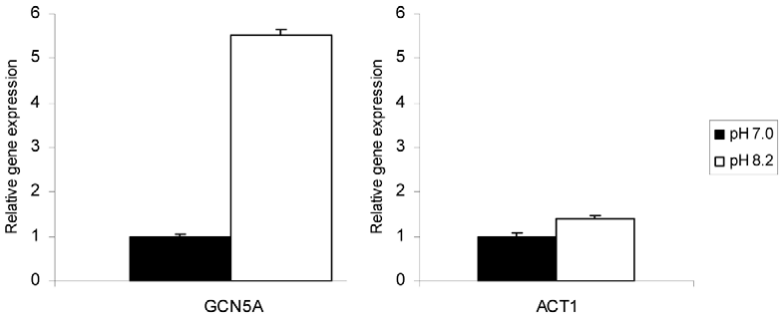
TgGCN5-A mRNA is up-regulated in response to alkaline stress. Wild-type (WT) parasites were incubated in control (pH 7.0) or alkaline media (pH 8.2) for three days. The levels of mRNA expression for TgGCN5-A or ACT1 were monitored by quantitative real-time PCR. Gene expression relative to control is shown. Error bars indicate the S.D.

We have previously created a null mutation of the TgGCN5-A gene by replacing the majority of the genomic locus with a hypoxanthine-xanthine-guanine phosphoribosyltransferase (HXGPRT) minigene in type I RH strain parasites lacking HXGPRT, designated ΔGCN5-A [21]. The loss of TgGCN5-A had no overt effect on tachyzoites grown in standard culture conditions [21], mirroring phenotypes reported for other lower eukaryotes such as yeast [14]. In other species, GCN5 has been shown to be important for stress responses and developmental changes [23]. In *Toxoplasma*, stress can lead to expression of bradyzoite-specific genes and the eventual formation of tissue cysts. We predicted that if TgGCN5-A played a role in stress-induced bradyzoite gene expression, then it may be up-regulated in response to a stress agent. After 3 days in alkaline culture conditions (pH 8.2), message levels for TgGCN5-A increase >5-fold in wild-type RH *Toxoplasma* (Fig. 1). Actin was monitored as a control to show that alkaline pH stress does not globally increase gene expression (Fig. 1).

To further assess if TgGCN5-A played a role in the stress response in *Toxoplasma* tachyzoites, we performed whole genome expression profiling to compare ΔGCN5-A and wild-type parasites grown for 3 days either in alkaline pH (8.2) medium or control medium. Gene profiling of certain *Toxoplasma* strains under stress has been reported previously, but the effects of alkaline pH stress on RH strain has not been examined [24]. Affymetrix ToxoGeneChip microarrays, which contain probe sets for ∼8000 predicted *Toxoplasma* genes, were used for this study. The differentially regulated genes were grouped into functional categories based on Gene Ontology (GO) annotations in the *Toxoplasma* genome database at ToxoDB.org. The entire dataset of *Toxoplasma* genes affected by alkaline pH stress is available as supplemental data (Dataset S1). TgGCN5-A was present in wild-type and absent in ΔGCN5-A, validating the identity of the samples and supporting the fidelity of the microarray analysis. The fidelity of select microarray results was further confirmed through independent qPCR (Table S1).

**Figure 2:**
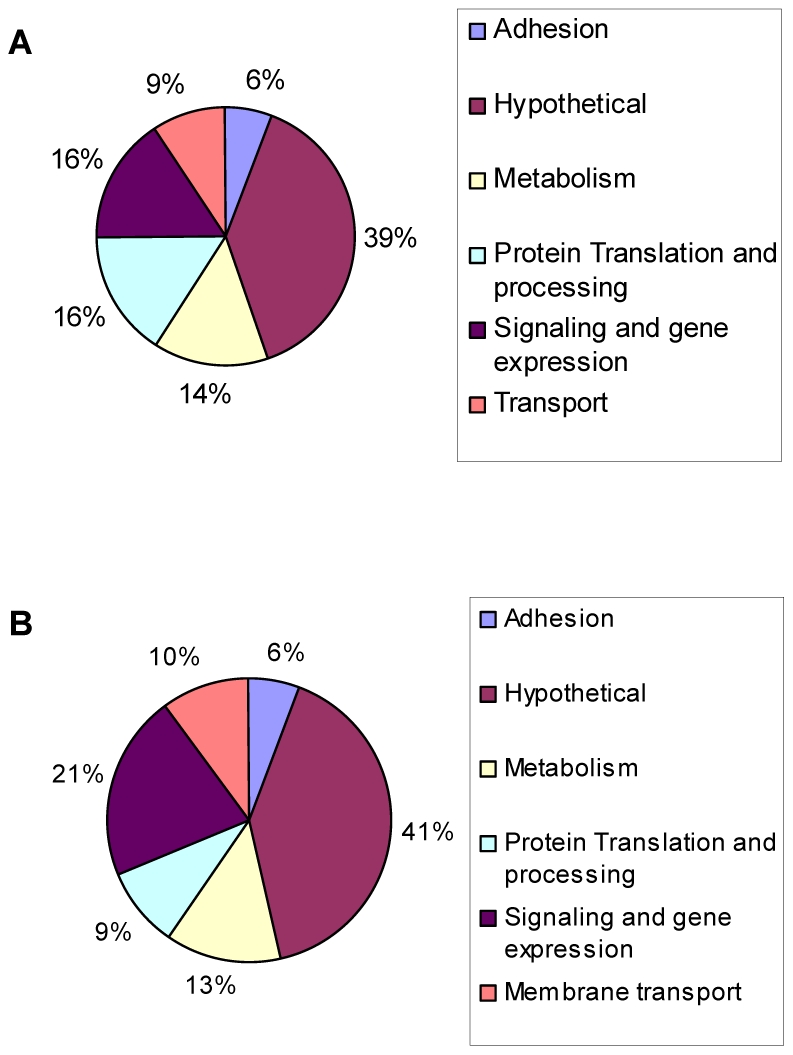
Genes modulated during alkaline pH stress in wild-type *Toxoplasma*. Microarray analysis was performed on wild-type parasites incubated in HFFs cells under normal culture conditions (pH 7.0) or alkaline conditions (pH 8.2) for three days. Pie chart displays the GO categories of genes reported to be (A) up-regulated or (B) down-regulated in response to alkaline conditions (p<0.001).

Results show that ∼14% (1,114) of genes are differentially regulated (p value<0.001) in intracellular wild-type parasites after 3 days of alkaline pH exposure. Similar to findings in *S. cerevisiae* [25], a broad range of genes have altered expression in *Toxoplasma* during alkaline pH exposure, including genes involved in invasion, metabolism, protein processing, signaling and gene expression, and membrane transport. Of the 1,114 genes affected, 592 genes were up-regulated (Fig. 2A) and 522 genes were down-regulated (Fig. 2B). Among the genes with largest changes in response to alkaline stress (an arbitrary cut-off of 2-fold or more), 177 were up-regulated (Table S2) and 84 were down-regulated (Table S3).

#### Common Environmental Response/Environmental Stress Response genes

Many of the genes altered during pH stress in *Toxoplasma* have been previously described as being part of the Common Environmental Response (CER) or Environmental Stress Response (ESR) [26], [27] while others appear specific to the alkaline stress response.

CER/ESR is characterized by increased expression of genes involved in a wide array of cellular processes including metabolism, detoxification, protein folding and degradation, metabolite transport, vacuolar and mitochondrial function, and intracellular signaling [26], [27]. Our genome-wide analysis revealed that intracellular tachyzoites exposed to alkaline pH have increased levels of messages encoding enzymes involved in glycogen synthesis, such as 1,4-alpha-glucan branching enzyme, glycan synthase, and other gluconeogenic enzymes that facilitate glycogen synthesis (Table S4). Moreover, the 2.3-fold increase in trehalose-6-phosphate synthase transcript suggests trehalose synthesis increases during alkaline stress in *Toxoplasma* (Table S4). Glycogen and trehalose act as stores of energy reserves, stabilize proteins, and provide osmolyte balance during stress [28], [29].

Another classical feature of CER/ESR is the up-regulation of protein folding enzymes and protein degrading enzymes. Our microarray study showed that pH-stressed tachyzoites generally have increased levels of chaperones and protein folding enzymes such as TCP-1/cpn chaperones and DnaJ domain containing proteins. Sixteen genes involved in protein folding increased while 7 showed reduced expression (Table S4 and Dataset S1). Similarly, the pH-stressed parasites up-regulate factors involved with proteosomal degradation, suggesting that they are preparing to remove malfolded proteins. During pH stress in *Toxoplasma*, ribosomal genes tend to be slightly depressed or unchanged, consistent with the cell shifting to an energy saving mode.

Another feature of CER/ESR is the induction of genes that provide protection from oxidizing agents, including transporters that maintain ion homeostasis and an intracellular reducing environment. In alkaline pH-stressed tachyzoites, thioredoxins, glutraredoxins, and glutathione reductase genes are up-regulated, as well as genes encoding a cation transporter, solute transporter, and sodium/hydrogen exchangers (Table S4). We conclude that tachyzoites exposed to alkaline stress exhibit many parallels to the CER/ESR reported in yeast [26], [27], supporting the idea that this type of core stress response arose very early in eukaryotic cell evolution.

#### pH-specific changes in gene expression

Very few studies have examined the alkaline stress response of eukaryotic cells in detail. Studies in *S. cerevisiae* have demonstrated up-regulation of several phosphate transport and metabolism genes in response to alkaline pH, including PHO84, PHO11/PHO12, and PHO89, as well as ion transport and homeostasis genes such as CTR3, FRE1, and ARN4 [27], [30], [31]. We found a potential homologue of PHO84 in *Toxoplasma* (46.m01634) that is increased nearly 2-fold in wild-type parasites exposed to alkaline stress. Our data also shows that a ctr copper transporter domain-containing protein increases 2-fold (55.m05007), two calcium-transporting ATPase increase ∼2-fold (44.m02594 and 583.m00010), and a cation-transporting ATPase (80.m00077) increases 2.8-fold.

A number of C2H2 zinc finger transcription factors are known to be up-regulated during alkaline pH exposure in *S. cerevisiae*, such as Rim101 and NRG2 [30]. Only one predicted C2H2 zinc finger protein was slightly up-regulated in *Toxoplasma*, 55.m04837 (1.3-fold), and it is not clear if it is orthologous to Rim101 or NRG2. TIS11 is a CCCH type zinc finger protein up-regulated during alkaline stress in yeast [30]; two zinc fingers of this class were up-regulated >2-fold in *Toxoplasma* exposed to alkaline stress. Genes encoding several other zinc finger proteins were also increased during alkaline stress (Table 1). A number of transcription factors in Apicomplexa containing plant-like Apetela-2 domains (AP2) also increased during alkaline pH stress; those increasing 2-fold or more include 20.m03817, 80.m02236, 37.m00767, and 641.m01483 (Table 2). No AP2 domain proteins were down-regulated 2-fold or more. The data suggest that the *Toxoplasma* and yeast alkaline stress response share some common features, but there are clearly parasite-specific changes taking place, as evidenced by the up-regulation of 75 hypothetical genes of unknown function at least 2-fold in alkaline pH-stressed *Toxoplasma* (Table S5).

**Table 1:** Predicted zinc finger proteins up-regulated during alkaline stress in *Toxoplasma.*

Accession no.	fold	p-value	Predicted function
64.m00581	6.68	0.033432	zinc finger MYND domain-containing protein
44.m02516	3.51	0.004565	zinc finger (CCCH type) protein, putative
20.m03752	2.70	0.016895	zinc finger (CCCH type) protein, putative
83.m00009	2.52	0.009715	DHHC zinc finger domain-containing protein
49.m03283	2.36	0.009751	B-box zinc finger protein, putative
59.m06100	2.14	0.001095	B-box zinc finger protein, putative
65.m00004	1.94	0.000260	zinc finger DHHC domain-containing protein
57.m01858	1.92	0.000034	zinc finger (C3HC4 RING finger) protein, putative
44.m04667	1.89	0.000901	CW-type zinc finger domain-containing protein
86.m00377	1.60	0.000655	zinc finger (CCCH type) protein, putative
55.m00100	1.56	0.012026	zinc finger (C3HC4 RING finger) protein, putative
74.m00769	1.5	0.002618	zinc finger (C3HC4 RING finger) protein, putative

*Table displays genes up-regulated 1.5-fold or more. See Dataset S1 for complete dataset.*

**Table 2:** Predicted AP2 proteins up-regulated during alkaline stress in *Toxoplasma.*

Accession no.	fold	p-value	Predicted function
20.m03817	5.35	0.000296	AP2 domain transcription factor VIIa-4 (AP2VIIa-4)
80.m02236	3.89	0.005110	AP2 domain transcription factor IX-5 (AP2IX-5)
37.m00767	3.35	0.001780	AP2 domain transcription factor XII-2 (AP2XII-2)
641.m01483	3.02	0.000227	AP2 domain transcription factor IV-4 (AP2IV-4)
52.m01583	1.85	0.005337	AP2 domain transcription factor III-2 (AP2III-2)
74.m00465	1.68	0.000244	AP2 domain transcription factor VIIa-8 (AP2VIIa-8)
20.m00367	1.59	0.001080	AP2 domain transcription factor VIIa-3 (AP2VIIa-3)
33.m01378	1.57	0.045700	AP2 domain transcription factor X-11 (AP2X-11)
33.m01343	1.54	0.000312	AP2 domain transcription factor X-9 (AP2X-9)

*Table displays genes up-regulated 1.5-fold or more. See Dataset S1 for complete dataset.*

TgGCN5-A itself was not observed to increase during alkaline stress by microarray analysis, appearing to contradict the highly reproducible findings in Fig. 1. However, only a small portion of the 5′ end of the TgGCN5-A gene is represented on the ToxoGeneChip array, which likely prevents accurate measurements of TgGCN5-A. In contrast, the qPCR primers used for Fig. 1 were designed to the 3′ end of the open reading frame, providing much more accurate measurements of TgGCN5-A message levels.

### Deficient gene regulation in ΔGCN5-A parasites during alkaline stress

**Figure 3:**
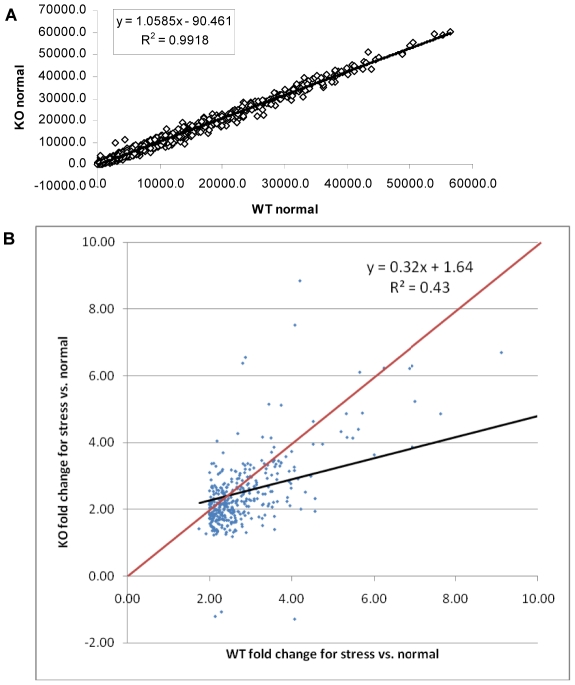
ΔGCN5-A parasites fail to up-regulate alkaline pH response genes. A. Scatter plot of expression levels for ΔGCN5-A (KO – vertical axis) vs. wild-type (WT –horizontal axis) under normal conditions. Data limited to those genes with fraction present ≥0.5 in WT or KO. B. Scatter plot of wild-type (WT) vs. ΔGCN5-A knockout (KO) fold changes. Probe sets limited to those with maximum fraction present ≥0.5, p<0.01 and fold ≥2.0 for WT stress vs. normal culture conditions. Regression line is in black. All points below the red line are genes with smaller fold changes in KO than in WT for stress vs. normal culture conditions.

**Figure 4:**
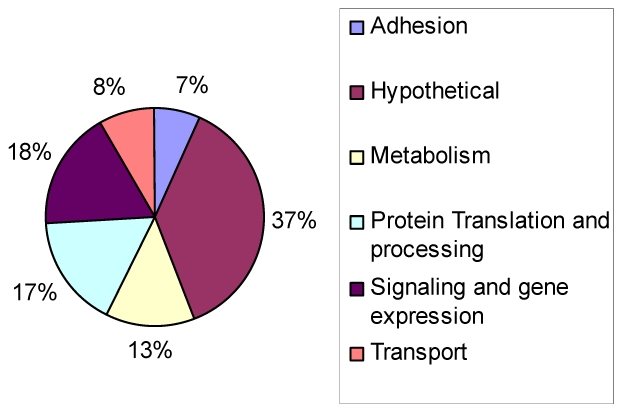
Types of genes modulated by TgGCN5-A during alkaline stress. Pie chart displays GO categories of genes that were not up-regulated in ΔGCN5-A parasites relative to wild-type (p<0.001) after culture in alkaline (pH 8.2) conditions.

A scatter plot of the microarray data reveals that there are virtually no differences (R^2^ = 0.99) in gene expression patterns between wild-type and ΔGCN5-A parasites grown under normal culture conditions (Fig. 3A), which is consistent with the indiscernible phenotype of ΔGCN5-A cultured in normal conditions. However, relative to wild-type, the ΔGCN5-A parasites differ dramatically in their ability to regulate gene expression when grown in alkaline medium (R^2^ = 0.43) (Fig. 3B). While 1,114 genes are altered in wild-type parasites exposed to alkaline stress, only 502 genes were changed in alkaline-stressed parasites lacking TgGCN5-A (p<0.001). Since TgGCN5-A is an activator of gene expression we focused on the genes up-regulated during alkaline stress. Further examination of up-regulated genes reveals that ΔGCN5-A parasites fail to up-regulate 439 of the 592 genes up-regulated by wild-type parasites grown in alkaline pH. In other words, TgGCN5-A is required for increased expression of 74% of the genes activated in response to alkaline pH. TgGCN5-A-dependent genes have diverse roles in signaling and gene expression (18%), protein processing and translation (17%), metabolism (13%), membrane transport (8%), and adhesion (7%) (Fig. 4). TgGCN5-A also controls a substantive number of hypothetical proteins with no known function (37%). Hypothetical genes with a change of 2-fold or more are listed in Table S5.

Our microarray data (p<0.05) identifies numerous genes related to Ca^2+^ signaling that were not up-regulated normally in parasites lacking TgGCN5-A. These include calcium-dependent protein kinase (86.m00003), calmodulin beta (42.m03474), calmodulin genes (50.m03141, 59.m03587), and calcium/calmodulin-dependent 3′, 5′-cyclic nucleotide phosphodiesterases (583.m05366, 59.m03644) (Dataset S1). Although the eukaryotic response to alkaline exposure is poorly understood, transient increases in intracellular calcium occur, possibly activating calcineurin and leading to a signaling cascade that results in mobilization of transcription factors [25], [31].

### TgGCN5-A is enriched at genes up-regulated during alkaline stress

**Figure 5:**
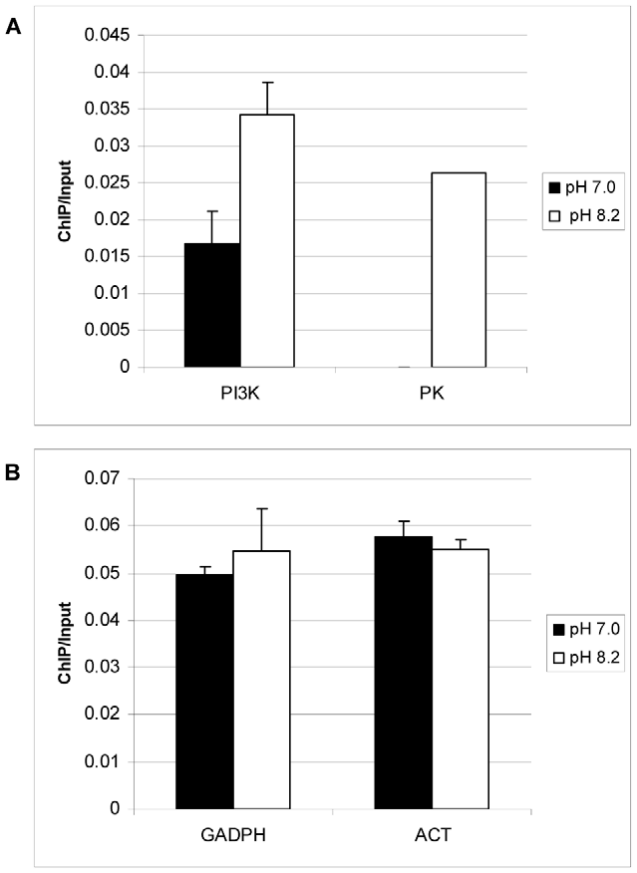
TgGCN5-A is enriched at genes up-regulated during alkaline pH stress. *Toxoplasma* parasites stably expressing FLAG-tagged TgGCN5-A were maintained in HFFs with normal (pH 7.0) or alkaline (pH 8.2) medium prior to harvesting for qChIP using anti-FLAG antibody. Immunoprecipitated DNA was examined using primers to pH responsive genes phosphatidylinositol 3- and 4-kinase (PI3K), and protein kinase (PK) (A), or housekeeping gene controls, GAPDH and actin, ACT (B). Results are represented as a ratio of ChIP/Input DNA. The difference between pH 7 and pH 8 for PI3K is statistically significant (p = 0.02, Student's t-test).

Previously we have demonstrated that increased acetylation accompanies TgGCN5-A promoter occupancy [3]. To obtain *in vivo* confirmation that TgGCN5-A plays a direct role in the co-activation of genes shown to be up-regulated during alkaline culture, we used chromatin immunoprecipitation (ChIP). RH parasites expressing FLAG-tagged TgGCN5-A (fGCN5-A) were employed in ChIP experiments to purify DNA in association with fGCN5-A using anti-FLAG [3], [32]. We examined a region ∼1.0 kb upstream of the start ATG for phosphatidylinositol 3- and 4-kinase (PI3-4K, 76.m01548) and protein kinase (PK, 641.m01507) genes, both of which are up-regulated during pH stress (p<0.05, Dataset S1). Two housekeeping genes, actin (25.m00007) and glyceraldehyde-3-phosphate dehydrogenase (GAPDH, 80.m00003), whose expression was not altered during pH stress, were included as controls. ChIP data show an enrichment of fGCN5-A at a region upstream of PI3K and PK during alkaline stress (Fig. 5A). The levels of fGCN5-A remained unchanged at housekeeping genes, demonstrating that the increase of fGCN5-A at pH-responsive genes above is not random (Fig. 5B). We performed an additional negative control for each ChIP sample using a nonspecific antibody (anti-TgIF2K-A) as described previously [33], none of which produced a signal (data not shown). Combined with the microarray analysis, these data support the idea that TgGCN5-A is required for proper gene activation in response to alkaline stress.

### ΔGCN5-A parasites fail to activate bradyzoite genes in response to alkaline stress

**Figure 6:**
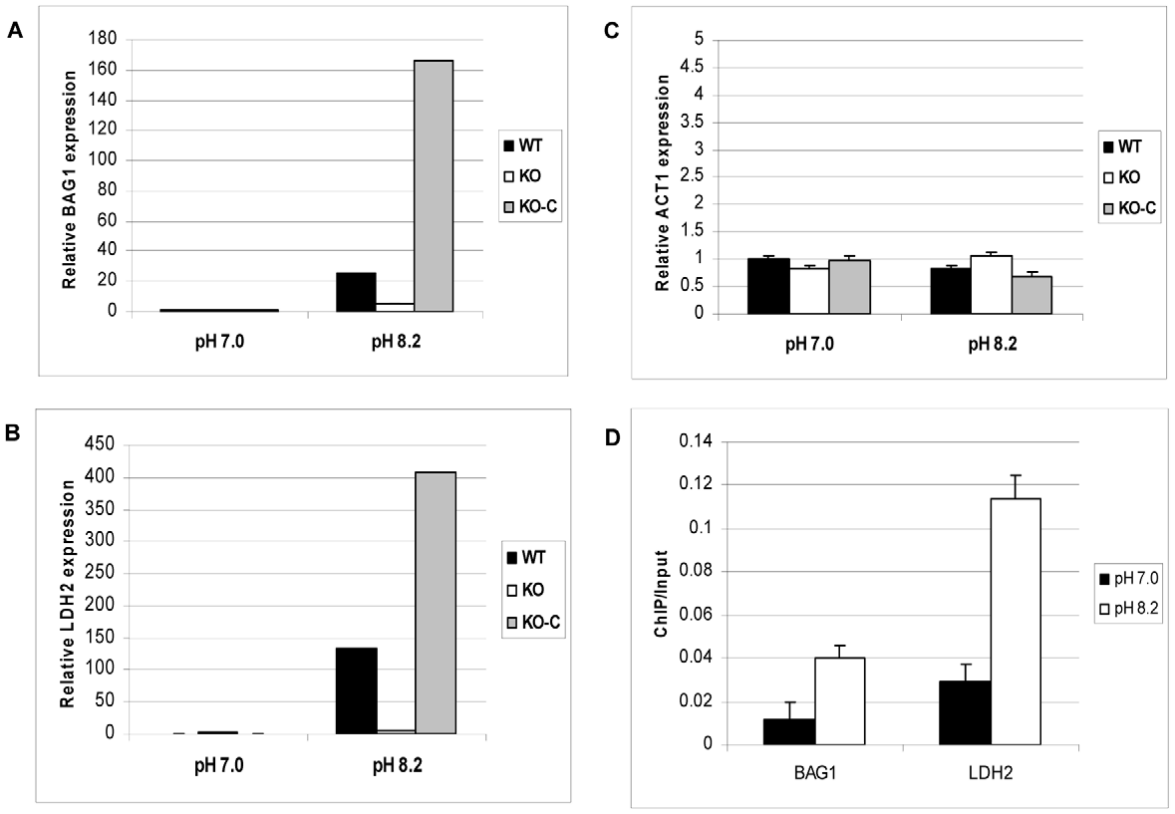
ΔGCN5-A parasites fail to up-regulate developmental genes in response to alkaline pH. Wild-type (WT), ΔGCN5-A (KO), or complemented ΔGCN5-A (KO-C) parasites were incubated in control (pH 7.0) or alkaline media (pH 8.2) for four days. The levels of mRNA expression for stress-induced bradyzoite genes BAG1 (A) and LDH2 (B) or the constitutive gene actin, ACT1, (C) were monitored by quantitative real-time PCR. Error bars indicate the S.D. D. ChIP of FLAG-tagged TgGCN5-A performed as described in Fig. 5, but using primers for BAG1 and LDH2 (Table S6). Results are represented as a ratio of ChIP/Input DNA. P value for BAG1 sample = 0.02, P value for LDH2 sample = 0.01 (Student's t-test).

The impaired response to alkaline pH stress suggests that ΔGCN5-A parasites may also be defective in bradyzoite development. The knockout was made in the hypervirulent type I RH strain, which does not fully develop into bradyzoites at high frequency. RH strain will generally not form cyst walls, but will express detectable levels of bradyzoite-specific marker genes in response to stresses that induce cyst formation [34]. In our hands, we can detect BAG1 and LDH2 mRNAs by day 4 of alkaline pH treatment. To test if TgGCN5-A plays a role in stress-induced bradyzoite gene expression, we grew wild-type or ΔGCN5-A parasites in pH 7.0 (control) or pH 8.2 (stress) media. At day 4, intracellular parasites were harvested for quantitative real-time PCR to monitor mRNA levels for bradyzoite-specific genes BAG1 and LDH2. While wild-type parasites up-regulate both bradyzoite marker genes in response to pH 8.2, the ΔGCN5-A parasites fail to do so (Fig. 6A and 6B). Actin was monitored as a control gene that does not significantly change during bradyzoite induction (Fig. 6C). To ensure the defect was not an indirect effect, we complemented ΔGCN5-A parasites by stably expressing a recombinant copy of TgGCN5-A. Expression of BAG1 and LDH2 was restored in the complemented ΔGCN5-A parasites exposed to alkaline stress (Fig. 6A and 6B). The mRNA levels of BAG1 and LDH2 following stress in complemented ΔGCN5-A parasites are higher than wild-type, presumably because the recombinant TgGCN5-A is being expressed above wild-type levels. Interestingly, this higher level of TgGCN5-A expression in the complemented clone does not affect levels of BAG1 or LDH2 under non-stressed conditions, implying that TgGCN5-A does not activate these bradyzoite genes until a stress signal is perceived by the parasite. We used ChIP analysis to further demonstrate the involvement of TgGCN5-A in regulating developmentally expressed genes. ChIP demonstrates that TgGCN5-A is recruited to BAG1 and LDH2 promoter regions during alkaline stress (Fig. 6D). We conclude that parasites lacking TgGCN5-A are defective in up-regulating bradyzoite-specific genes in response to alkaline stress.

### TgGCN5-A knockout parasites are deficient in recovery from alkaline stress

Based on our results, it would be predicted that parasites exposed to alkaline stress would have difficulty recovering from the insult. To test this hypothesis, intracellular parasites were exposed to pH 8.2 for 3 or 5 days, harvested, and then inoculated into fresh host cells and cultured under normal (pH 7.0) conditions. Parasite proliferation was monitored using the PCR-based B1 assay. Data show that intracellular parasites lacking GCN5-A exposed to pH 8.2 for 3 days do not recover as efficiently as wild-type or the GCN5-A complemented clone (Fig. S1). The recovery defect is even more pronounced when the intracellular ΔGCN5-A parasites are subjected to pH 8.2 for 5 days (Fig. S1).

**Figure 7:**
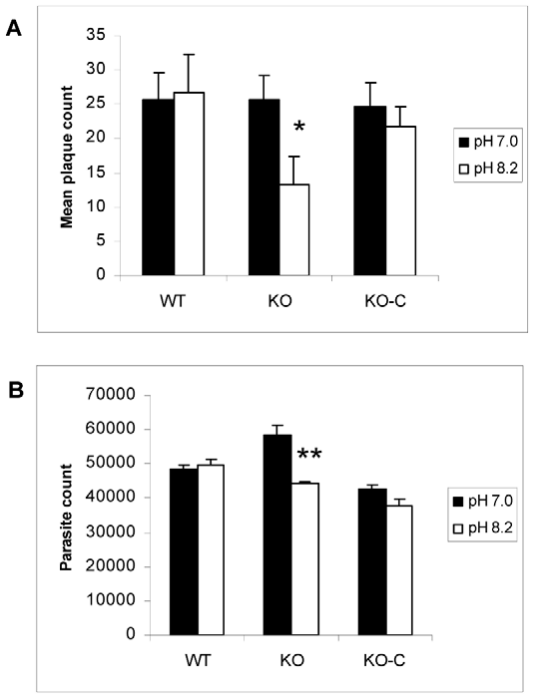
Parasites lacking TgGCN5-A are defective in recovering from alkaline stress. Equal numbers of extracellular wild-type (WT), ΔGCN5-A (KO), or complemented ΔGCN5-A (KO-C) parasites were subjected to alkaline (pH 8.2) or control (pH 7) media for 30 min at 37°C in 5% CO_2_. Following treatment, the parasites were allowed to infect HFF monolayers and incubated under normal culture conditions. A. Parasite growth was monitored with a standard plaque assay at day 5 (* denotes p = 0.017). B. Viability assay was set up as described above, but growth was monitored with the PCR-based B1 assay (** denotes p = 0.012).

The preceding studies were performed on tachyzoite-infected host cells. We also examined if direct exposure to alkaline stress produced a phenotype in the parasites. In order to test if alkaline pH impacts ΔGCN5-A parasites directly, we monitored the ability of purified, extracellular ΔGCN5-A tachyzoites to recover from a short term exposure to alkaline pH. Equal numbers of extracellular ΔGCN5-A or wild-type tachyzoites were placed in media of pH 7.0 (control) or 8.2 for 30 min, and then allowed to infect confluent host cell monolayers under normal culture conditions. At day five, plaques in the infected monolayers were counted. The ΔGCN5-A mutant displayed increased sensitivity to alkaline pH as evidenced by its impaired ability to produce plaques following this insult (Fig. 7A). Recombinant TgGCN5-A was able to restore the ability of ΔGCN5-A parasites to recover from alkaline stress (Fig. 7A). We verified the results with a second, independent type of growth assay that monitors parasite proliferation through quantitative PCR of the parasite-specific B1 gene (Fig. 7B). Collectively, these studies establish that TgGCN5-A is a key factor that manages the *Toxoplasma* response to alkaline pH stress in RH strain tachyzoites. It is curious that the complemented clone, which appears to be over-expressing TgGCN5-A, does not offer greater protection from alkaline stress despite being able to up-regulate BAG1 and LDH2 greater than wild-type (Fig. 6). GCN5 HATs function in large multi-subunit complexes, the components of which vary in different cells or under different conditions. A possible explanation could be that a “minimal” GCN5 complex can up-regulate certain stress response genes, but it is not capable of providing greater protection to the cell because other components are required that are not over produced.

**Figure 8:**
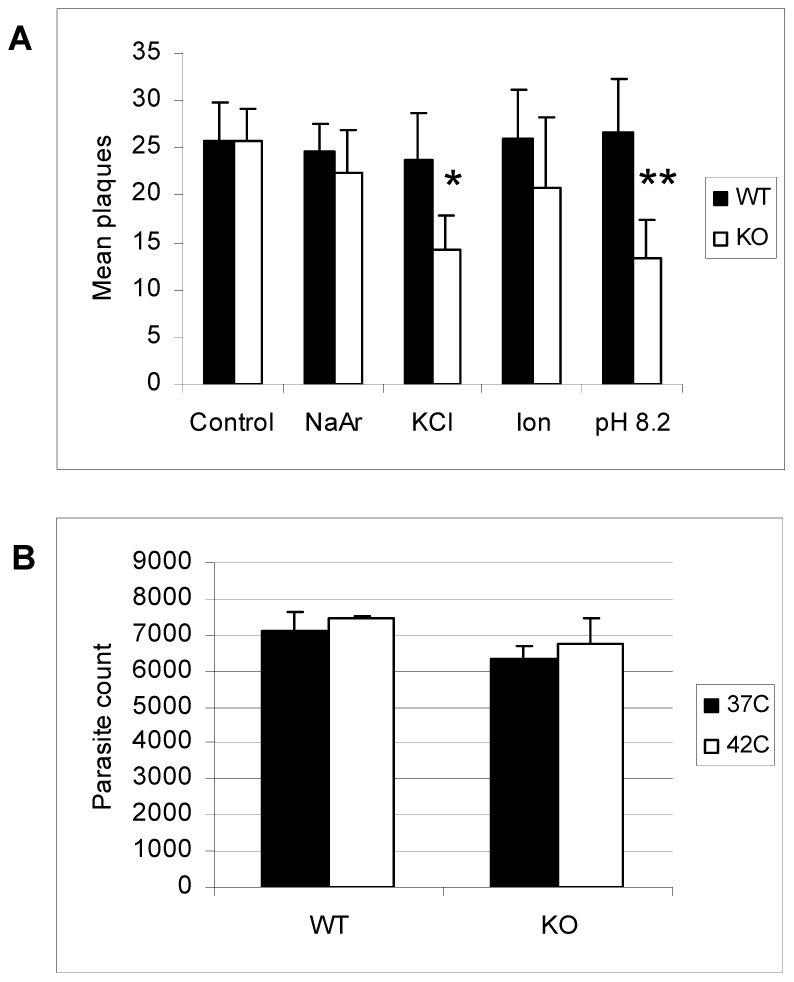
Response of ΔGCN5-A parasites to other stress conditions. A. Equal numbers of extracellular wild-type (WT) or ΔGCN5-A (KO) were subjected to one of the following stresses for 30 min before being placed back into HFF culture under normal growth conditions: 0.6 M KCl, 5 µM sodium arsenite (NaAr), or 1 µM ionophore (Ion). Alkaline pH (8.2) stress was used as a positive control and no stress as a negative control. The mean number of plaques after five days recovery in HFF monolayer culture is shown. P values for Student's t-test are denoted as * = 0.03 and ** = 0.02. B. Equal numbers of extracellular wild-type (WT) or ΔGCN5-A (KO) were incubated at 37°C or 42°C for 30 minutes then placed in HFF culture at 37°C. The number of parasites in culture at day 5 post-infection was determined using the PCR-based B1 assay.

We examined whether ΔGCN5-A parasites were hypersensitive to other stresses, including 30 minute exposure to 0.6 M KCl, 5 µM arsenite, 1 µM ionophore, or 42°C heat shock. Upon being placed back in culture following the exposure to these stresses, the ΔGCN5-A parasites grew similarly to wild-type with exception of those exposed to KCl stress (Fig. 8A and 8B). Remarkably, TgGCN5-A appears to have a striking specificity for managing the alkaline and possibly KCl stress responses, possibly because each disrupts ion potential. Such narrow specificity in stress response has been reported for *Schizosaccharomyces pombe* GCN5 [15].

### Concluding remarks

*Toxoplasma gondii* possesses two GCN5 KATs, which is unusual as lower eukaryotes tend to have a single GCN5. We have sought to delineate the roles of these two GCN5s by making genetic knockouts. The ΔGCN5-A mutant is viable and does not show growth defects under normal culture conditions; however, attempts to generate a knockout of TgGCN5-B have not been successful. These results support a model that TgGCN5-B is essential for housekeeping functions while TgGCN5-A is required to overcome certain stress situations. Such a role for TgGCN5-A is established by the studies described herein. It remains possible that there is some functional overlap between the two TgGCN5s, but whatever contribution is made by TgGCN5-B is not sufficient to compensate for the loss of TgGCN5-A in terms of responding normally to alkaline pH stress, including the up-regulation of bradyzoite marker genes BAG1 and LDH2.

A key finding in this study is that TgGCN5-A is required to activate developmental genes in response to pH stress. These studies were performed in type I RH strain, which is not well suited for a more thorough characterization of bradyzoite development *in vitro* or *in vivo*. Such studies would require the generation of an analogous TgGCN5-A knockout in type II strain *Toxoplasma*. Our initial attempts to disrupt the TgGCN5-A knockout in type II strains have not yet succeeded, probably due to technical challenges inherent in working with the slow growing type II strain, but it is possible that TgGCN5-A is essential in type II strain.

It is intriguing that *Toxoplasma* possesses a duplicate GCN5 HAT that appears to be exquisitely tailored to respond to alkaline, and to a lesser extent, KCl stress. Adaptation to fluctuations in pH is likely to be relevant to proliferating tachyzoites, as pH stress almost certainly is encountered by *Toxoplasma in vivo* as it moves in and out of host cells throughout diverse regions of the body. Additionally, while not addressed for *Toxoplasma* infection, it has been reported that other infections elevate intracellular pH [35]. It is difficult to distinguish whether the observed changes in intracellular parasites are due to direct effects of high pH on *Toxoplasma* parasites themselves or indirect effects produced by the response of host cells. What is clear is that RH strain parasites lacking TgGCN5-A are defective in regulating changes in the transcriptome that accompany the response to alkaline pH. The data are significant as alkaline pH is considered a “gold standard” method for triggering bradyzoite development *in vitro* [1]. In summary, our studies establish that TgGCN5-A plays a major role in the normal response to alkaline pH stress, including the activation of developmentally regulated genes, in *Toxoplasma* RH strain. The conclusion is based on multiple lines of data from independent studies, including up-regulation of TgGCN5-A mRNA during pH stress, microarray analysis, TgGCN5-A enrichment at genes up-regulated during alkaline stress, and phenotypic analysis showing that the TgGCN5-A knockout has impaired alkaline stress recovery. Our microarray analysis also provides novel insight into the molecular basis of the alkaline stress response in intracellular *Toxoplasma*.

## Materials and Methods

### Parasite culture and reagents

*Toxoplasma* tachyzoites (wild-type (WT) RH, ΔGCN5-A, and TgGCN5-A complemented lines) were cultured in primary human foreskin fibroblasts (HFF) using Dulbecco's Modified Eagle's Medium (DMEM) supplemented with 1.0% fetal bovine serum (FBS, Invitrogen). Parasites were grown in a humidified CO_2_ (5%) incubator at 37°C. Cultures were confirmed to be free of *Mycoplasma* contamination by MycoAlert Assay (Cambrex Bio Science). Parasites were harvested immediately following lysis of host cell monolayers and purified by filtration through a 3.0 micron filter [36]. Bradyzoite growth conditions were identical except the infection medium was replaced with alkaline medium (DMEM with 20 mM HEPBS, 2 g NaHCO_3_/L and 1.0% FBS, adjusted to pH 8.2 using NaOH) and changed daily.

### Stress conditions and parasite growth assays

Tachyzoites were released from HFF monolayers using a 25 gauge syringe needle, and filtered to remove the host cell debris was spun out at 500×*g* for 5 min. Extracellular parasites were resuspended at a concentration of 10^5^ parasites/ml in DMEM, alkaline medium (pH 8.2, adjusted with NaOH as described above), or medium containing 0.6 M KCl, 5 µM arsenite, 1 µM ionophore. This suspension was incubated at 37°C (or 42°C for heat shock experiment) in 5% CO_2_ for 30 min, and then 10^3^ parasites were inoculated onto HFF monolayers in 24-well plates. After 5 day incubation at 37°C, the infected monolayers were fixed with 100% methanol and stained with crystal violet to score the number of plaques [36] or processed for B1 PCR assay [37].

### RNA preparation and hybridization to microarrays

Confluent HFF monolayers grown in T25-cm^2^ flasks were infected with 10^6^ parasites using normal parasite culture media (above). After 2 hr, infection medium was replaced with normal medium (pH 7.0) or alkaline medium (see above). Flasks were maintained in humidified 37°C incubator in 5% CO_2_. Medium for both normal and alkaline cultures was replaced each day for 3 days, at which point the infected monolayers were scraped with a rubber policeman. Samples were centrifuged (1500 rpm, 10 min) and resuspended in sterile PBS. Intracellular parasites were released from host cells by syringe passage using a 25 gauge needle and washed in PBS. Total RNA was isolated from the purified parasites using an RNeasy Mini Kit according to the manufacturer's instructions (Qiagen). To minimize genomic DNA contamination, additional treatment with DNase was performed. The cDNA and cRNA were synthesized according to the protocols recommended by Affymetrix in their GeneChip Expression Analysis Technical Manual (Affymetrix, Santa Clara, CA). Briefly, cDNA was synthesized using T7 promoter-dT24 oligonucleotide as primer with the Invitrogen Life Technologies SuperScript Choice system. Biotinylated cRNA was made using the Affymetrix *in vitro* transcription (IVT) labeling kit. Fifteen µg of biotinylated cRNA was added to a total hybridization cocktail of 300 µl, and 200 µl was hybridized to an Affymetrix custom *T. gondii* ToxoGeneChip (http://roos-compbio2.bio.upenn.edu/~abahl/Array-Tutorial.html) after adding control oligonucleotides. Hybridization was performed at 45°C for 17 h with constant rotation. The hybridization mixture was then removed and the GeneChips were washed, stained with phycoerythrin-labeled streptavidin, washed, incubated with biotinylated anti-streptavidin, and re-stained with phycoerythrin-labeled streptavidin to amplify the signals; these steps were carried out using the Affymetrix Fluidics Station. To reduce non-random error, balanced groups of samples were handled in parallel. Arrays were then scanned using the dedicated scanner, controlled by Affymetrix GeneChip Operating Software (GCOS). The Affymetrix Microarray Suite version 5 (MAS5) algorithm was used to analyze the hybridization intensity data from GeneChip expression probe arrays and to calculate the set of metrics that describe probe set performance. The average intensity on each array was normalized by global scaling to a target intensity of 1000. For each of the two conditions (normal and alkaline), four independent preparations of WT and ΔGCN5-A RNA were prepared; each of the 16 preparations was hybridized to its own microarray to ensure a strong statistical basis for analysis.

### Statistical analysis and data deposition

We analyzed only those probe sets (genes) that were called “present” by the MAS5 detection call in at least half of the arrays for at least one of the four conditions to eliminate probe sets that are not reliably detected (those at or near background or that may reflect cross-hybridization) [38]. We used a Welch's t-test on log_2_ transformed MAS5 signals to reveal significant differences between WT and ΔGCN5-A. The resultant p-values were used to calculate false discovery rates (FDR) using the Benjamini and Hochberg method [39]. Fold-changes were calculated by taking the ratio of the mean of the WT and ΔGCN5-A signal values, using the larger mean as the numerator; by convention we show the result as negative if the mean of the ΔGCN5-A samples was smaller. Microarray data are available at Gene Expression Omnibus (http://www.ncbi.nlm.nih.gov/geo/), accession number GSE22100.

### Real-time reverse transcription-PCR (qRT-PCR)

Primers were designed using the Primer Express 2.0 software (Applied Biosystems, CA) and are listed in Table S6. The RT reaction was performed using 1.0 µg total RNA isolated from designated tachyzoites, with oligo-dT primers and Omniscript reverse transcriptase according to the manufacturer's directions (Qiagen). 1.0 µl of a 1∶10 dilution of the resulting cDNA was amplified in a 25 µl total volume containing SYBR Green PCR Master Mix (Applied Biosystems, CA) and 0.5 µM of each forward and reverse primer. All reactions were performed in triplicate using the 7500 Real-time PCR system and analyzed with relative quantification software (7500 software v2.0.1) (Applied Biosystems, CA). The *Toxoplasma* β-tubulin gene (Genbank accession number M20025) was used to normalize the qRT-PCRs. β-tubulin was determined to be equivalent between samples using actin and GAPDH as normalizing controls.

### Chromatin immunoprecipitation (ChIP)

ChIP was performed on tachyzoites stably expressing fGCN5-A using polyclonal anti-FLAG antibody (Sigma F7425) immobilized to Dynabeads Protein A (Invitrogen). Quantitative PCR was performed as described above. Immunoprecipitated DNA samples were quantified using a standard curve created with serially diluted input DNA. 0.1 ng of total ChIP DNA was added to each reaction and reactions were performed in triplicate. Primers used are listed in Table S6, each pair designed to amplify ∼90 bp regions located ∼1.0 kb upstream of the ATG start site.

### Generation of complemented ΔGCN5-A parasite clones

To complement ΔGCN5-A parasites, the ptubXFLAG::HX *Toxoplasma* expression vector [32] was modified to replace its HX minigene marker with a CAT minigene to confer resistance to chloramphenicol. Recombinant, tagged full-length TgGCN5-A was cloned into the vector using the NdeI and AvrII sites, referred to as ptub_MYC_GCN5-A_FLAG_::CAT. The TgGCN5-A coding sequence was amplified from *Toxoplasma* cDNA using Phusion® High-Fidelity DNA Polymerase (New England Biolabs) and primers containing NdeI and AvrII restriction enzyme sites (italicized below). The sense primer contained sequence encoding the MYC epitope tag (underlined) and the antisense primer lacked the TgGCN5-A stop codon to allow in-frame fusion with a FLAG tag in the vector [32]: sense, 5′-ATACCAT*CATATG*AAAATGGCGTACCCGTACGACGTCCCGGACTACGCGGAGACTGTCGAAGTGCCTGCATTC; antisense, 5′-ATACCAT*CCTAGG*GAAACTCCCGAGAGCCTCGACCTTGGGCC. 10^6^ ΔGCN5-A parasites were transfected with 20 µg NotI-linearized vector and selected for resistance to 20 µM chloramphenicol before cloning by limiting dilution as previously described [36]. Multiple clones were selected and verified to express ectopic _MYC_GCN5-A_FLAG_ protein by IFA using anti-FLAG (Sigma F7425). Phenotypes reported were similar for multiple independent clones.

## Supporting Information

Figure S1 Impact of alkaline pH stress on intracellular parasites. Cultures of wild-type (WT, black), ΔGCN5-A (KO, white), or complemented ΔGCN5 (KO-C, gray) parasites were subjected to alkaline media for 3 (top panel) or 5 (lower panel) days. Parasites were harvested from host cells and then allowed to infect fresh host cells under normal culture conditions (pH 7.0). Parasites were allowed to grow for 5 days and then quantitated using the PCR-based B1 assay. Day 3 WT vs KO p = 0.02 and day 5 WT vs KO p = 0.001, using student's t-test.
(0.27 MB PDF)

Dataset S1 Microarray dataset of differential gene expression in wild-type and ΔGCN5-A parasites grown in alkaline pH (8.2) or control (pH 7.0) medium for three days. Data for three p values (0.001, 0.01, and 0.05) are provided.
(2.49 MB XLS)

Table S1 Independent RT-PCR confirmation of select microarray results. We examined 4 genes up-regulated, 4 genes down-regulated, and 4 genes unchanged in the knockout relative to wild-type following the alkaline stress. In each case, the trend in gene expression as measured by RT-PCR was similar to that reported in the microarray data.
(0.05 MB PDF)

Table S2 Transcripts that are induced >2-fold in *Toxoplasma* grown in alkaline medium.
(0.06 MB PDF)

Table S3 Genes down-regulated 2-fold or more in response to alkaline stress (p<0.001).
(0.01 MB PDF)

Table S4 CER/ESR genes up-regulated during *Toxoplasma* response to alkaline stress (p<0.05).
(0.01 MB PDF)

Table S5 Hypothetical genes up-regulated during alkaline pH stress in *Toxoplasma* (p<0.001).
(0.01 MB PDF)

Table S6 List of primers used in this study.
(0.01 MB PDF)
