# Supplementary figures and images for: Toxoplasma gondii Lysine Acetyltransferase GCN5-A Functions in the Cellular Response to Alkaline Stress and Expression of Cyst Genes
Source: PLoS Pathog. 2010 Dec 16;6(12):e1001232. doi: 10.1371/journal.ppat.1001232 (PMC3003489; doi:10.1371/journal.ppat.1001232)

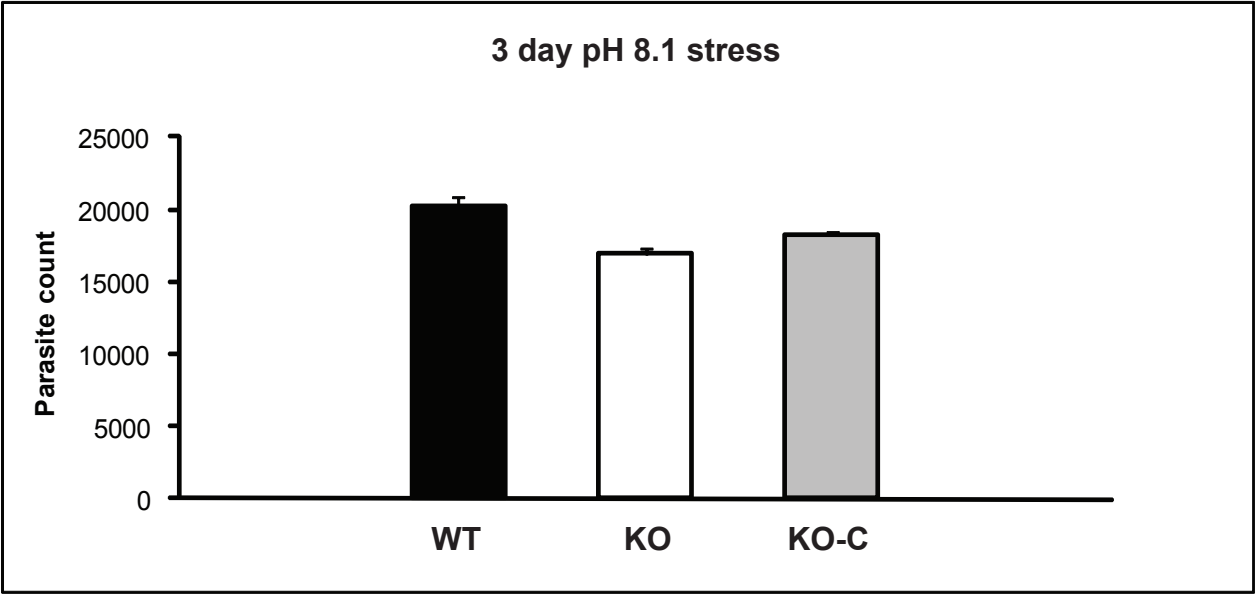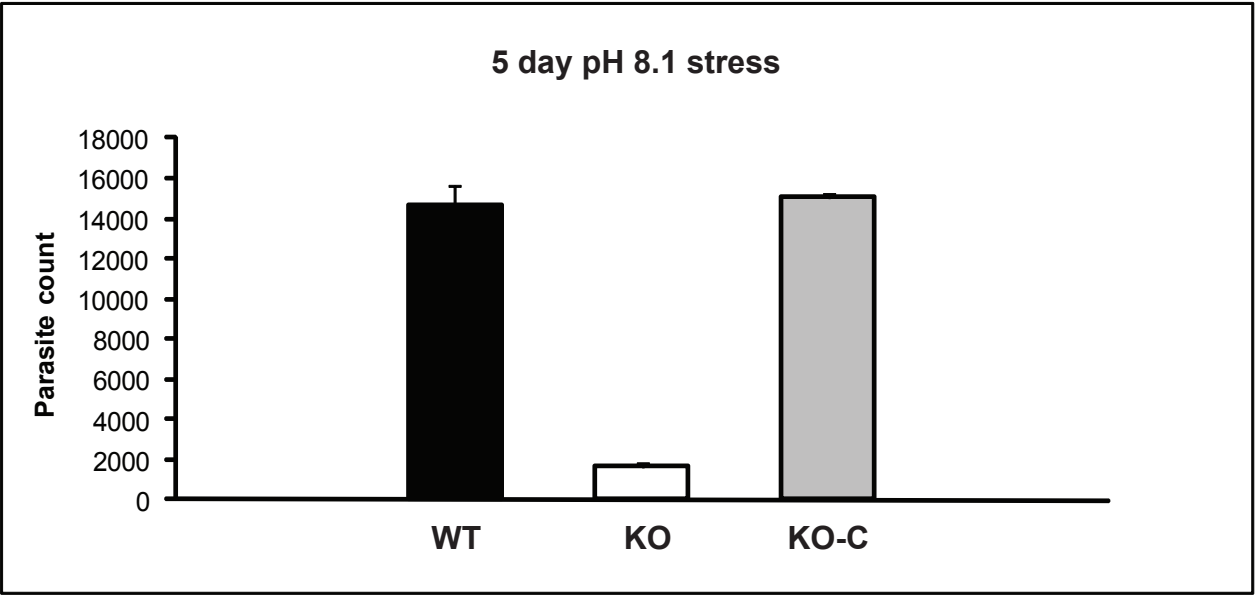

Supplement: Figure S1 — Impact of alkaline pH stress on intracellular parasites. Cultures of wild-type (WT, black), ΔGCN5-A (KO, white), or complemented ΔGCN5 (KO-C, gray) parasites were subjected to alkaline media for 3 (top panel) or 5 (lower panel) days. Parasites were harvested from host cells and then allowed to infect fresh host cells under normal culture conditions (pH 7.0). Parasites were allowed to grow for 5 days and then quantitated using the PCR-based B1 assay. Day 3 WT vs KO p = 0.02 and day 5 WT vs KO p = 0.001, using student's t-test. (0.27 MB PDF) [file ppat.1001232.s001.pdf]
