# Supplementary material for: Toxoplasma gondii Lysine Acetyltransferase GCN5-A Functions in the Cellular Response to Alkaline Stress and Expression of Cyst Genes
Source: PLoS Pathog. 2010 Dec 16;6(12):e1001232. doi: 10.1371/journal.ppat.1001232 (PMC3003489; doi:10.1371/journal.ppat.1001232)
Supplement: Table S1 — Independent RT-PCR confirmation of select microarray results. We examined 4 genes up-regulated, 4 genes down-regulated, and 4 genes unchanged in the knockout relative to wild-type following the alkaline stress. In each case, the trend in gene expression as measured by RT-PCR was similar to that reported in the microarray data. (0.05 MB PDF) [file ppat.1001232.s003.pdf]

Table S1: Independent RT-PCR confirmation of microarray data

	Microarray		RT-PCR	
Gene ID	WT	ΔGCN5-A	WT	ΔGCN5-A
Genes up-regulated in WT during alkaline stress				
641.m01566	2.29	-1.06	1.5±0.03	-1.4±0.01*
541.m01237	3.29	1.52	2.4±0.05	1.3±0.08
641.m01498	7.4	2.0	2.8±0.08	1.3±0.1
76.m01548	7.01	5.24	-2.8±0.04	-3.6±0.09
Genes with no significant change in WT during alkaline stress				
41.m02959	1.35	2.05	1.07±0.07	1.5±0.7
49.m03159	1.2	2.38	-1.1±0.03	1.8±0.14
162.m00002	1.16	2.4	-1.25±0.06	1.4±0.6
641.m01597	-1.05	1.2	-2.1±0.03	-1.6±0.17*
Genes down-regulated in WT during alkaline stress				
20.m00368	-4.37	-3.7	-4.7±0.03	-8.3±0.09
46.m01600	-3.22	-2.74	-6.2±0.05	-3.7±0.09
55.m04769	-6.6	-6.31	-1.2±0.1	-4.5±0.1
641.m01514	-3.21	-3.24	-15.6±0.1	-3.8±0.3

Values represent fold-change in microarrays and fold change normalized to tubulin mRNA levels in RT-PCRs (±S.E. ΔCt). WT = wild-type. Student's t-test was performed for RT-PCR data and p values were <0.05 except for two samples indicated with asterisks. With exception of 76.m01548, each gene tested reproduces the trend observed with the microarrays.
