# Supplementary material for: Toxoplasma gondii Lysine Acetyltransferase GCN5-A Functions in the Cellular Response to Alkaline Stress and Expression of Cyst Genes
Source: PLoS Pathog. 2010 Dec 16;6(12):e1001232. doi: 10.1371/journal.ppat.1001232 (PMC3003489; doi:10.1371/journal.ppat.1001232)
Supplement: Table S2 — Transcripts that are induced >2-fold in Toxoplasma grown in alkaline medium. (0.06 MB PDF) [file ppat.1001232.s004.pdf]

Table S2. Transcripts that are induced >2-fold in *Toxoplasma* grown in alkaline medium

Accession no.	Fold	Predicted function
Adhesion & Invasion		
162.m00311	6.94	SRS domain-containing protein
583.m05680	5.62	SRS domain-containing surface antigen, putative
63.m00337	4.16	SRS47E
162.m00313	3.75	GPI-anchored surface antigen, putative
**59.m03665**	**3.52**	**SRS domain-containing surface antigen, putative**
25.m01768	3.50	kinesin motor domain-containing protein
**25.m00004**	**3.08**	**SAG2 related antigen SAG2B**
55.m04865	2.56	microneme protein, putative
**64.m00327**	**2.37**	**membrane skeletal protein IMC1-related**
**641.m01566**	**2.29**	**GPI-anchored surface antigen, putative**
**52.m01567**	**2.24**	**cell wall protein-related**
**49.m03223**	**2.12**	**myosin light chain kinase, putative**
Metabolism		
**44.m02535**	**77.19**	**haloacid dehalogenase-like hydrolase domain containing protein**
**541.m01207**	**5.33**	**dihydrolipoamid dehydrogenase, putative**
**50.m00029**	**4.06**	**1,4-alpha-glucan branching enzyme, putative**
**542.m00223**	**3.74**	**thymidylate kinase, putative**
**44.m00012**	**3.94**	**3-oxoacyl-synthase, putative / acyl-carrier-protein, putative**
**41.m01331**	**3.68**	**phosphoglycerate kinase, putative**
**83.m00003**	**3.51**	**ribonucleoside-diphosphate reductase, large subunit, putative**
**541.m01237**	**3.29**	**oxidoreductase, putative**
**59.m03618**	**3.14**	**transketolase, putative**
76.m01567	3.10	pyruvate carboxylase, putative
**49.m03154**	**3.00**	**serine:pyruvate/alanine:glyoxylate aminotransferase, putative**
50.m03138	2.86	acylamino-acid-releasing enzyme, putative
**44.m02770**	**2.85**	**deoxyuridine 5'-triphosphate nucleotidohydrolase, putative**
41.m00032	2.82	aldehyde dehydrogenase, putative
**641.m00168**	**2.67**	**malate dehydrogenase, putative**
**46.m01716**	**2.49**	**haloacid dehalogenase-like hydrolase domain-containing protein**
551.m00229	2.44	6-phosphogluconate dehydrogenase, putative
**80.m00049**	**2.42**	**opine dehydrogenase, putative**
**57.m01793**	**2.41**	**lysine decarboxylase domain-containing protein**
**57.m00028**	**2.34**	**isocitrate dehydrogenase, putative**
44.m02574	2.30	acetyltransferase domain-containing protein
**57.m01784**	**2.29**	**glycosyltransferase sugar-binding region containing DXD motif**
55.m05019	2.29	NADH-cytochrome B5 reductase, putative
59.m03723	2.12	thiF family domain-containing protein
**59.m03459**	**2.11**	**biotin carboxyl carrier protein, putative**
**583.m05389**	**2.10**	**apyrase, putative**
**50.m00016**	**2.05**	**dihydrofolate reductase-thymidylate synthase**
49.m03432	2.03	glucoamylase S1/S2 precursor-related
**551.m00022**	**2.03**	**ATP-binding protein-related**
**49.m05731**	**2.01**	**UDP-N-acetylglucosamine-1-phosphate transferase, putative**
**55.m00168**	**2.01**	**ATP synthase beta chain, putative**
Protein translation and processing		
**59.m07783**	**3.58**	**cysteine protease domain containing protein**
**55.m04698**	**2.95**	**prefoldin subunit 3, putative**
**50.m03182**	**2.92**	**glutaredoxin-related domain-containing protein**
**59.m03538**	**2.75**	**poly [ADP-ribose] polymerase-1, putative**
**583.m05330**	**2.60**	**PEANUT1**
76.m01670	2.44	peroxiredoxin family protein/glutaredoxin, putative
583.m09175	2.38	proteasome subunit beta type 3, putative
**49.m05715**	**2.34**	**OTU-like cysteine protease domain-containing protein**
**83.m01219**	**2.06**	**poly(ADP)-ribose polymerase-related**
38.m00013	2.06	histidyl tRNA synthetase 2
**55.m05059**	**2.05**	**ubiquitin carboxyl-terminal hydrolase, putative**
**641.m00192**	**2.02**	**TCP-1/cpn60 family chaperonin, putative**
**52.m00007**	**2.01**	**proteasome subunit beta type 2, putative**

**39.m00356**	**2.00**	**lysyl-tRNA synthetase-related**
Signaling and gene expression		
**20.m03817**	**5.35**	**AP2 protein**
**46.m01736**	**3.71**	**replication factor C small subunit, putative**
65.m01169	3.64	D4 dopamine receptor-related
**52.m01667**	**3.57**	**protein kinase-related**
**39.m00361**	**3.45**	**3'5'-cyclic nucleotide phosphodiesterase, putative**
113.m00762	3.41	MORN repeat-containing protein
**641.m00011**	**3.14**	**proliferating cell nuclear antigen 2, putative**
641.m01483	3.02	AP2 protein
**49.m00049**	**2.96**	**DNA replication licensing factor, putative**
**50.m03076**	**2.86**	**origin recognition complex subunit 2-related**
**20.m00342**	**2.82**	**replication factor A-related protein, putative**
**38.m01060**	**2.69**	**cyclin-dependent kinase-related**
**42.m00103**	**2.67**	**DNA-binding protein HU, putative**
**38.m01077**	**2.53**	**DNA replication licensing factor, putative**
**49.m05649**	**2.52**	**bolA-like protein-related**
80.m02294	2.42	NUDIX domain-containing protein
**50.m03192**	**2.40**	**ATPase 2, putative**
**46.m01743**	**2.25**	**protein kinase-related**
59.m03392	2.24	DNA polymerase alpha subunit-related
50.m00018	2.23	adenosine kinase
**50.m03356**	**2.22**	**caltractin (centrin), putative**
**35.m00895**	**2.18**	**EF hand domain-containing protein**
**44.m02816**	**2.17**	**DNA polymerase delta small subunit, putative**
**59.m03702**	**2.16**	**TGF-beta resistance-associated protein-related**
55.m04912	2.16	DNA mismatch repair protein, putative
55.m04926	2.16	histone H2A, putative
**49.m03276**	**2.13**	**protein kinase domain-containing protein**
20.m03683	2.12	TPR domain-containing protein
**59.m03368**	**2.11**	**phosphoinositide-dependent protein kinase, putative**
49.m03212	2.10	maf-like protein, putative
**52.m01606**	**2.05**	**importin-alpha re-exporter, putative**
**52.m01539**	**2.04**	**DNA polymerase epsilon p17 subunit, putative**
Transport		
**50.m00011**	**3.62**	**enoyl-acyl carrier reductase**
**113.m00769**	**3.38**	**membrane transporter PFB0465c**
**80.m00077**	**2.84**	**cation-transporting ATPase, putative**
**55.m08201**	**2.38**	**vacuolar protein sorting 26, putative**
**55.m00019**	**2.29**	**acyl carrier protein**
49.m03236	2.23	L-type amino acid transporter-related
25.m01818	2.21	mitochondrial carrier domain-containing protein
541.m01185	2.19	ABC transporter, putative
**26.m00235**	**2.09**	**lava lamp protein-related / peripheral golgi protein-related**
**52.m01549**	**2.04**	**vacuolar protein sorting 29, putative**
641.m01548	2.04	L-type amino acid transporter-related
**44.m02594**	**2.01**	**calcium-transporting ATPase, putative**
**50.m05656**	**2.01**	**mitochondrial carrier domain-containing protein**

See Table S5 for list of hypothetical genes (49 of 75 not up-regulated in ΔGCN5-A)
Entries in **bold** are not up-regulated in parasites lacking TgGCN5-A. Genes with $p<0.001$ are displayed.
