# Supplementary material for: Toxoplasma gondii Lysine Acetyltransferase GCN5-A Functions in the Cellular Response to Alkaline Stress and Expression of Cyst Genes
Source: PLoS Pathog. 2010 Dec 16;6(12):e1001232. doi: 10.1371/journal.ppat.1001232 (PMC3003489; doi:10.1371/journal.ppat.1001232)
Supplement: Table S3 — Genes down-regulated 2-fold or more in response to alkaline stress (p<0.001). (0.01 MB PDF) [file ppat.1001232.s005.pdf]

Table S3. Genes down-regulated 2-fold or more in response to alkaline stress (p<0.001)

Accession no.	Fold	Product description
Adhesion and Invasion		
25.m01745	-2.05	tubulin delta chain, putative
35.m00027	-2.67	ankyrin repeat-containing protein
20.m03764	-2.94	cell wall protein-related
641.m01514	-3.21	SRS domain-containing protein
46.m01600	-3.22	surface antigen, putative
55.m04769	-6.60	sporozoite-specific SAG protein
Hypothetical		
113.m00813	-2.73	hypothetical protein
145.m00330	-3.98	hypothetical protein
145.m00583	-2.06	hypothetical protein
20.m03768	-2.02	hypothetical protein
25.m01812	-2.24	hypothetical protein
33.m01282	-2.39	hypothetical protein, conserved
33.m01326	-2.51	hypothetical protein
33.m01360	-3.60	hypothetical protein
37.m00005	-3.22	hypothetical protein
37.m00011	-2.13	hypothetical protein
39.m00359	-2.06	hypothetical protein
41.m02947	-2.18	hypothetical protein
42.m03410	-2.33	hypothetical protein
42.m03640	-2.19	hypothetical protein
44.m02540	-2.23	hypothetical protein
44.m02742	-2.01	hypothetical protein, conserved
49.m03124	-2.12	hypothetical protein
49.m03176	-2.02	hypothetical protein
49.m03257	-2.19	hypothetical protein
49.m05663	-2.39	hypothetical protein
50.m03090	-3.23	hypothetical protein
50.m05602	-2.01	hypothetical protein
50.m07108	-3.09	hypothetical protein
50.m07651	-2.40	hypothetical protein, conserved
50.m07652	-2.06	hypothetical protein
52.m00026	-2.83	hypothetical protein
52.m02726	-3.39	hypothetical protein
52.m03461	-2.53	hypothetical protein, conserved
541.m01242	-5.91	hypothetical protein
57.m01859	-2.36	hypothetical protein
57.m03133	-2.16	hypothetical protein
583.m05626	-2.13	hypothetical protein
583.m05630	-2.33	hypothetical protein
583.m05736	-2.06	hypothetical protein
583.m09170	-2.01	hypothetical protein
588.m00009	-2.38	hypothetical protein
59.m03370	-3.70	hypothetical protein
59.m03419	-3.17	hypothetical protein
59.m03529	-2.60	hypothetical protein

59.m03623	-2.20	hypothetical protein
59.m07778	-2.48	hypothetical protein, conserved
64.m00341	-2.06	hypothetical protein
644.m00074	-2.67	hypothetical protein
65.m01106	-3.73	hypothetical protein
65.m01170	-2.53	hypothetical protein
65.m01969	-3.05	hypothetical protein
69.m00262	-3.17	hypothetical protein
76.m01629	-2.19	hypothetical protein
76.m01634	-3.87	hypothetical protein
77.m00087	-3.54	hypothetical protein
80.m02124	-2.81	hypothetical protein
83.m00006	-2.05	hypothetical protein
83.m00011	-2.16	hypothetical protein
83.m02139	-2.47	hypothetical protein
86.m00371	-2.48	hypothetical protein
86.m00395	-2.13	hypothetical protein
Metabolism		
35.m00933	-2.36	ATPase, AFG1 family domain-containing protein
49.m00045	-2.17	bcs1 protein, putative
55.m08207	-2.51	carbonic anhydrase-related
Miscellaneous		
20.m00337	-2.09	vitamin K epoxide reductase complex subunit 1, putative
44.m02688	-2.64	ppg3, putative
80.m02212	-2.24	white protein, putative
Protein translation & processing		
20.m00387	-2.09	subtilase family serine protease, putative
20.m03958	-2.16	insulinase-related
76.m02661	-2.21	glutathione S-transferase, putative
50.m03181	-2.50	leucine rich repeat protein, putative
Signaling and gene expression		
80.m05396	-2.30	serine/threonine-protein kinase, putative
42.m00006	-2.28	serine/threonine protein phosphatase, putative
80.m00073	-2.79	ruvB-like 2, putative
49.m05665	-2.45	protein kinase, putative
25.m02928	-2.24	phosphatidylethanolamine-binding protein homolog, putative
80.m02249	-2.29	N-acetyltransferase 5, putative
49.m05708	-2.21	G-protein beta-subunit (transducin), putative
50.m03277	-2.18	endonuclease/exonuclease/phosphatase domain-containing protein
42.m03509	-2.41	calmodulin-domain protein kinase, putative
Transport		
20.m00368	-4.37	transmembrane domain-containing protein
641.m01580	-2.78	outer membrane protein romA
83.m02142	-2.52	transmembrane domain-containing protein
