# Supplementary material for: Toxoplasma gondii Lysine Acetyltransferase GCN5-A Functions in the Cellular Response to Alkaline Stress and Expression of Cyst Genes
Source: PLoS Pathog. 2010 Dec 16;6(12):e1001232. doi: 10.1371/journal.ppat.1001232 (PMC3003489; doi:10.1371/journal.ppat.1001232)
Supplement: Table S4 — CER/ESR genes up-regulated during Toxoplasma response to alkaline stress (p<0.05). (0.01 MB PDF) [file ppat.1001232.s006.pdf]

Table S4. CER/ESR genes up-regulated during *Toxoplasma* response to alkaline stress ($p<0.05$)

Accession no.	Fold	P value	Product description
Glycogen and trehalose			
50.m00029	4.06	3.6×10^{-4}	1,4-alpha-glucan branching enzyme, putative
41.m01331	3.68	2.1×10^{-4}	phosphoglycerate kinase, putative
76.m01567	3.10	5.4×10^{-4}	pyruvate carboxylase, putative
113.m00792	2.34	1.3×10^{-2}	trehalose-6-phosphate synthase domain-containing protein
59.m00091	1.92	8.1×10^{-5}	glyceraldehyde-3-phosphate dehydrogenase
Protein folding			
55.m04698	2.95	1.3×10^{-5}	prefoldin subunit 3, putative
50.m03182	2.92	1.5×10^{-5}	glutaredoxin-related domain-containing protein
49.m03081	2.47	2.1×10^{-3}	thioredoxin, putative
76.m01670	2.44	2.2×10^{-4}	peroxiredoxin family protein/glutaredoxin, putative
641.m00192	2.02	1.5×10^{-4}	TCP-1/cpn60 family chaperonin, putative
26.m00247	2.01	1.2×10^{-2}	DnaJ domain-containing protein
49.m00030	1.93	2.6×10^{-5}	TCP-1/cpn60 family chaperonin, putative
77.m00088	1.82	1×10^{-5}	TCP-1/cpn60 family chaperonin, putative
540.m00198	1.68	4.7×10^{-2}	glutaredoxin domain containing protein
50.m00069	1.68	9×10^{-5}	thioredoxin, putative
50.m00003	1.64	5.2×10^{-5}	glutathione reductase, putative
55.m04854	1.62	1.8×10^{-2}	DnaJ domain-containing protein
583.m00697	1.58	1.1×10^{-3}	prefoldin subunit 5, putative
59.m03713	1.57	5.6×10^{-4}	10 kDa chaperonin, putative
44.m02569	1.56	9×10^{-6}	TCP-1/cpn60 family chaperonin, putative
80.m02201	1.54	4×10^{-4}	prefoldin subunit, putative
Proteosome degradation			
50.m03405	2.90	1.8×10^{-2}	ubiquitin-conjugating enzyme E2, putative
583.m09175	2.38	1.1×10^{-4}	proteasome subunit beta type 3, putative
55.m05059	2.05	4.7×10^{-4}	ubiquitin carboxyl-terminal hydrolase, putative
52.m00007	2.01	7.5×10^{-5}	proteasome subunit beta type 2, putative
50.m03375	2.01	3.4×10^{-3}	26S proteasome regulatory particle non-ATPase subunit-related
72.m00688	1.97	1.2×10^{-4}	proteasome A-type and B-type domain-containing protein
42.m00098	1.80	1.5×10^{-5}	26S proteasome regulatory ATPase subunit, putative
72.m00386	1.78	2.8×10^{-3}	ubiquitin conjugation factor-related
583.m05554	1.78	9.5×10^{-5}	26S proteasome non-ATPase regulatory subunit 2, putative

49.m03152	1.76	6.9×10^{-5}	proteasome subunit alpha type 4, subunit
57.m01709	1.70	7.6×10^{-3}	ubiquitin-activating enzyme-related
35.m00882	1.69	3.9×10^{-4}	proteasome subunit alpha type 3, putative
50.m00017	1.65	1.4×10^{-5}	proteasome subunit alpha type 5, putative
38.m01078	1.63	1.9×10^{-5}	proteasome subunit beta type 5, putative
41.m00016	1.60	3.4×10^{-2}	ubiquitin carboxyl-terminal hydrolase, putative
25.m00217	1.57	3.3×10^{-5}	proteasome PCI domain-containing protein
86.m00396	1.57	2.2×10^{-3}	ubiquitin-conjugating enzyme E2, putative
583.m05407	1.55	2.2×10^{-3}	ubiquitin carboxyl-terminal hydrolase, putative
44.m00017	1.54	2.4×10^{-4}	26S protease regulatory subunit 6b, putative
