# Supplementary material for: Toxoplasma gondii Lysine Acetyltransferase GCN5-A Functions in the Cellular Response to Alkaline Stress and Expression of Cyst Genes
Source: PLoS Pathog. 2010 Dec 16;6(12):e1001232. doi: 10.1371/journal.ppat.1001232 (PMC3003489; doi:10.1371/journal.ppat.1001232)
Supplement: Table S5 — Hypothetical genes up-regulated during alkaline pH stress in Toxoplasma (p<0.001). (0.01 MB PDF) [file ppat.1001232.s007.pdf]

Table S5. Hypothetical genes up-regulated during alkaline pH stress in *Toxoplasma*

Gene ID	Fold change
49.m03407	2.00
**25.m01855**	**2.01**
**49.m07231**	**2.01**
**57.m01783**	**2.01**
**33.m01307**	**2.04**
**42.m03322**	**2.05**
641.m00181	2.05
80.m03967	2.05
**49.m03332**	**2.06**
**83.m02696**	**2.06**
**57.m01745**	**2.08**
541.m02062	2.10
**31.m00932**	**2.11**
**583.m00704**	**2.11**
583.m05758	2.12
44.m02570	2.16
50.m05676	2.16
**55.m04796**	**2.16**
**583.m05299**	**2.17**
583.m05570	2.17
113.m00801	2.19
**76.m01587**	**2.20**
**57.m01870**	**2.24**
**44.m02673**	**2.25**
**44.m02788**	**2.27**
55.m10283	2.27
**49.m03114**	**2.28**
44.m02731	2.30
**49.m07196**	**2.31**
59.m03696	2.31
**49.m05689**	**2.32**
**49.m05666**	**2.36**
645.m00546	2.36
27.m00081	2.37
**641.m02570**	**2.37**
**52.m01558**	**2.38**
50.m03094	2.43
**583.m05687**	**2.46**

Gene ID	Fold change
**20.m05948**	**2.53**
44.m05934	2.53
**583.m05449**	**2.53**
**583.m05675**	**2.53**
**55.m10273**	**2.54**
55.m10281	2.57
**41.m01356**	**2.59**
44.m02600	2.59
**57.m01743**	**2.63**
55.m08192	2.68
**641.m01515**	**2.68**
**80.m02180**	**2.68**
37.m00739	2.69
59.m06055	2.75
**583.m05578**	**2.77**
**74.m00977**	**2.84**
80.m02121	2.86
**80.m02341**	**2.87**
**583.m09133**	**2.89**
**113.m01584**	**2.92**
**20.m00378**	**2.98**
**59.m06123**	**3.09**
**38.m01089**	**3.13**
**33.m02193**	**3.29**
**55.m04948**	**3.37**
**49.m03088**	**3.42**
**20.m05883**	**3.49**
**49.m03376**	**3.58**
**641.m01523**	**3.58**
**49.m03153**	**4.07**
46.m02888	4.08
55.m00162	4.20
**541.m01238**	**4.47**
65.m01150	4.57
**38.m01050**	**4.75**
83.m01238	5.35
37.m00740	7.64

*Entries in **bold** are not up-regulated in parasites lacking TgGCN5-A. Genes with p<0.001 are displayed.*
