# Supplementary material for: Toxoplasma gondii Lysine Acetyltransferase GCN5-A Functions in the Cellular Response to Alkaline Stress and Expression of Cyst Genes
Source: PLoS Pathog. 2010 Dec 16;6(12):e1001232. doi: 10.1371/journal.ppat.1001232 (PMC3003489; doi:10.1371/journal.ppat.1001232)
Supplement: Table S6 — List of primers used in this study. (0.01 MB PDF) [file ppat.1001232.s008.pdf]

Table S6. Primers used in this study

Primer sequence (5'-3')	Primer name	Use
TGAGCGAGTGTCCGGTTATTT	TgBAG1 RT F	Gene expression
TAGAACGCCGTTGTCCATTG	TgBAG1 RT R	Gene expression
ACAATGGCCCAGGCATTCT	TgLDH2 RT F	Gene expression
CAATAAACATATCGTGAAGCCCATA	TgLDH2 RT R	Gene expression
ATGTTCCGTGGTCGCATGT	Tub RT F	Gene expression
TGGGAATCCACTGAACGAAGT	Tub RT R	Gene expression
GGGCGGTTTCATGACCTAAA	TgACT1 RT F	Gene expression
ACGTATGATGCGCGAGAAAA	TgACT1 RT R	Gene expression
ATTTTGCTTGGGATTCGAGGA	GAPDH F	Gene expression
TGCAGGGTAACGATCAAAAAATG	GAPDH R	Gene expression
GGCTTCAGCAGCAAGATAACG	TgGCN5-A RT F	Gene expression
TGCTGAGACGACACTCCATCA	TgGCN5-A RT R	Gene expression
ACGAAGTTCAGTTGATGTTCAAGAA	TgGCN5-B RT F	Gene expression
GTTACGGAACTTGTCCAGCTCGTT	TgGCN5-B RT R	Gene expression
TCCTCTCCTGGATCTTCCTCC	Q-ChIP BAG F	Q-ChIP
TTGCACAAAACTGCCAAAGG	Q-ChIP BAG R	Q-ChIP
AAGTGTGCACGCTTTGCAAG	Q-ChIP LDH2 F	Q-ChIP
CATGCTCCGGGCAGTACCT	Q-ChIP LDH2 R	Q-ChIP
GAAATTTGGCGTTGACTGGC	Q-ChIP PK F	Q-ChIP
AGGACGGACTTTACCGGAAGA	Q-ChIP PK R	Q-ChIP
CTCACCAAGCTCGGAGTTCG	Q-ChIP PI3K F	Q-ChIP
TCCTTCTCTTCCACGGTCGA	Q-ChIP PI3K R	Q-ChIP
GCCATAAGCAATCGAACTGAGG	Q-ChIP ACT1 F	Q-ChIP
CGATAGGTCCAGCCGTGTGT	Q-ChIP ACT1 R	Q-ChIP
GCAGCGATCCGACTTTTTTT	Q-ChIP GADPH F	Q-ChIP
GCAGCGAAACAGTACCAGCAT	Q-ChIP GADPH R	Q-ChIP
GGAGGACTGGCAACCTGGTGTCG	Toxo B1-F	B1 growth assay
TTGTTTCACCCGGACCGTTTAGCAG	Toxo B1-R	B1 growth assay
GCTCTGCTCACGGGTTCAAA	162.m00002 F	array verification
CATTCGCTTCTGGACCAGGT	162.m00002 R	array verification
TGAAGCTAACAGTCCCCGAGA	641.m01566 F	array verification
CCGATGTCTGAGGGTTCGTC	641.m01566 R	array verification
TTTGGATGGAGACTCGGCAC	49.m03159 F	array verification
TTAACCGGGTCAGGCATGTT	49.m03159 R	array verification
AGAACGCCAACGAGCTCAAG	641.m01498 F	array verification
TTCTCATTGTCGTGTCCCTCC	641.m01498 R	array verification
GATAAGCGATCCGGCCAGT	41.m02959 F	array verification
GTGCGTATTTCCAAGGCGTC	41.m02959 R	array verification
TCTGAATCGGATCGGAGAGC	541.m01237 F	array verification
TTGATGACCTGGCCGGTAAT	541.m01237 R	array verification
GTCACAGTCCATCGCCATTG	20.m00368F	array verification
AAGAAACCGATGACCCCTCC	20.m00368R	array verification
TCGTGTGGTTCAGTTCCATCC	46.m01600F	array verification
TGGCATTGTTGCATTCGATC	46.m01600R	array verification
ACGAAGCCGCATTGAAAGAT	55.m04769F	array verification
CCGTCAGCGTGAAATCGTTT	55.m04769R	array verification
GAATGGTCGGGCTATGCGT	76.m01548F	array verification
TGCTAATCCGGTAGAGGCGT	76.m01548R	array verification

ATACTCGATGGCGCTCCTGT	641.m01514F	array verification
GCACGCTTTCTTCTCGCTGT	641.m01514R	array verification
